# Area 5 Influences Excitability within the Primary Motor Cortex in Humans

**DOI:** 10.1371/journal.pone.0020023

**Published:** 2011-05-16

**Authors:** Azra Premji, Navjot Rai, Aimee Nelson

**Affiliations:** Department of Kinesiology, University of Waterloo, Waterloo, Ontario, Canada; Indiana University, United States of America

## Abstract

In non-human primates, Brodmann's area 5 (BA 5) has direct connectivity with primary motor cortex (M1), is largely dedicated to the representation of the hand and may have evolved with the ability to perform skilled hand movement. Less is known about human BA 5 and its interaction with M1 neural circuits related to hand control. The present study examines the influence of BA 5 on excitatory and inhibitory neural circuitry within M1 bilaterally before and after continuous (cTBS), intermittent (iTBS), and sham theta-burst stimulation (sham TBS) over left hemisphere BA 5. Using single and paired-pulse TMS, measurements of motor evoked potentials (MEPs), short interval intracortical inhibition (SICI), and intracortical facilitation (ICF) were quantified for the representation of the first dorsal interosseous muscle. Results indicate that cTBS over BA 5 influences M1 excitability such that MEP amplitudes are increased bilaterally for up to one hour. ITBS over BA 5 results in an increase in MEP amplitude contralateral to stimulation with a delayed onset that persists up to one hour. SICI and ICF were unaltered following TBS over BA 5. Similarly, F-wave amplitude and latency were unaltered following cTBS over BA 5. The data suggest that BA 5 alters M1 output directed to the hand by influencing corticospinal neurons and not interneurons that mediate SICI or ICF circuitry. Targeting BA 5 via cTBS and iTBS is a novel mechanism to powerfully modulate activity within M1 and may provide an avenue for investigating hand control in healthy populations and modifying impaired hand function in clinical populations.

## Introduction

Excitatory and inhibitory neural circuitry within the primary motor cortex (M1) influence the neural output directed to the hand [1], and abnormalities in such circuitry may underlie impaired hand control in patient populations [2]–[5]. Neural circuitry within M1 is modified following repetitive transcranial magnetic stimulation (TMS) applied directly over M1 [6], [7] or to anatomically connected loci such as the premotor cortex [8]. Identifying novel neural paths to modify the output of M1 presents an opportunity to alter the neural circuits that underpin hand control. Such paths may serve as targets for TMS or other therapeutic regimes. The present study is focused on the influence of Brodmann's area 5 (BA 5) located in the medial superior parietal lobule (SPL) on the neural circuitry within M1.

In non-human primates, BA 5 significantly contributes to the control of hand movement [9]. BA 5 is largely dedicated to the upper limb and hand [10], [11], is well-differentiated in species with opposable thumbs and poorly defined or absent in those lacking this function suggesting a key role for its involvement in fine hand control [10], [11]. Receptive fields encompass the entire hand or several digits [11], [12] unilaterally or bilaterally [13], [14] and may be involved in the integration of somatic inputs between the hands [10]. Anatomical [15], [16] and electrophysiological [17] studies reveal direct projections from BA 5 to M1 with the magnitude of input as substantial [15] or greater [18] than that from the primary somatosensory cortex (SI). In humans, activity within BA 5 area of the SPL is enhanced during tactile motion discrimination [19], preparatory signals for upcoming finger-pointing [20], finger tracking [21], imagined finger movements [22], reaching and grasping [23], and bilaterally during tactile discrimination of objects [24]. The superior longitudinal fasciculus (SLF), an association fiber pathway, likely mediates the connectivity between BA 5 and ipsilateral M1 in humans [25] and monkeys [26].

Despite the anatomical connectivity, little is known about the functional importance of BA 5 to M1 interaction. In humans, TMS to area 5 facilitates the output of M1 during vibrotactile stimulation to the thumb and index fingers compared to rest [27]. One important question is whether area 5 influences the inhibitory and excitatory neural circuitry within M1. With the known role of BA 5 in hand control it is likely that this area imposes an important influence on the M1 neural circuitry underpinning motor control of the hand. Neural circuitry within M1 may be probed using single-pulse TMS using the amplitude of the resultant motor evoked potential (MEP) which reflects both cortical and spinal excitability. Circuitry may also be probed using paired-pulse TMS whereby two stimuli are delivered in rapid succession to the motor representation of a particular muscle [28]. Using this technique, the MEP is reduced at intervals between 1–6 ms [28]–[30] and enhanced at 8–30 ms [28], [30] reflecting short interval intracortical inhibition (SICI) and intracortical facilitation (ICF), respectively. The present study investigates the functional influence of BA 5 on the neural circuitry within M1.

Theta-burst stimulation is a repetitive TMS protocol that when delivered in continuous mode over M1 (cTBS) reduces MEPs [6], [7], [31], [32], ICF [6], SICI [6], [7] and alters the neural circuitry within contralateral M1 although the direction of the latter changes are variable [7], [31], [33]. In contrast, when delivered intermittently (iTBS), MEP amplitude and SICI increase [6], [7]. Further, cTBS to premotor cortex decreases MEP amplitude and has no effect on SICI or ICF [8] suggesting that select M1 neural circuitry may be modulated from remote areas. In the present studies we examined the modulation of inhibitory and excitatory neural circuits within M1 bilaterally following cTBS and iTBS over left hemisphere BA 5. We hypothesized that cTBS would decrease excitability within ipsilateral M1 resulting in a decrease in SICI, MEPs, and ICF, in parallel with changes observed following cTBS over M1 [6], [7], [33] and premotor cortex [8] and that iTBS would produce opposite effects in line with previous reports [6], [7]. To further elucidate the neural mechanisms of the BA 5 to M1 interaction, we investigated the influence of BA 5 on spinal excitability. To achieve this, F-waves from bilateral FDI muscles were recorded before and for up to one hour following cTBS over BA 5. Spinal excitability was not hypothesized to alter following cTBS since corticospinal projections from BA 5 are confined to the dorsal horn of the spinal grey matter [34], [35]. The large cortical representation of the hand [10], connectivity to M1 [15], [16] and role in skilled hand movement [11] suggest that BA 5 may modulate M1 output and may therefore be an important target for altering the control of hand movement.

## Methods

### Participants

The experiments were approved by the Office of Research Ethics at the University of Waterloo and conformed to the *Declaration of Helsinki*. Twenty-eight healthy participants were studied. Subjects were determined to be healthy using a 23 point TMS screening form that queried medical conditions. Twenty-four subjects participated in Experiment 1. Eleven subjects participated in Experiment 2. Ten participants were tested in both Experiment 1 and 2 and these experiments were separated by at least one week. Seven subjects participated in Experiment 3, four of whom participated in Experiment 1. Right-handedness was confirmed using a subset of the Edinburgh Handedness Inventory [36]. All subjects gave informed written consent prior to participation.

### Electromyographic (EMG) recording

Surface EMG was recorded from the first dorsal interosseous (FDI) muscle on the right and left hand using 9 mm diameter Ag-AgCl surface electrodes. The active electrodes were placed over the muscle belly and the reference electrode was placed over the metacarpophalangeal joint of the index finger. EMG was amplified 1000×, band-pass filtered between 2 Hz to 2.5 kHz (Intronix Technologies Corporation Model 2024F, Canada), digitized at 5 kHz by an analog-to-digital interface (Micro1401, Cambridge Electronics Design, Cambridge, UK) and stored on a computer for off-line analysis.

### Neuronavigation and Transcranial magnetic stimulation

**Figure 1:**
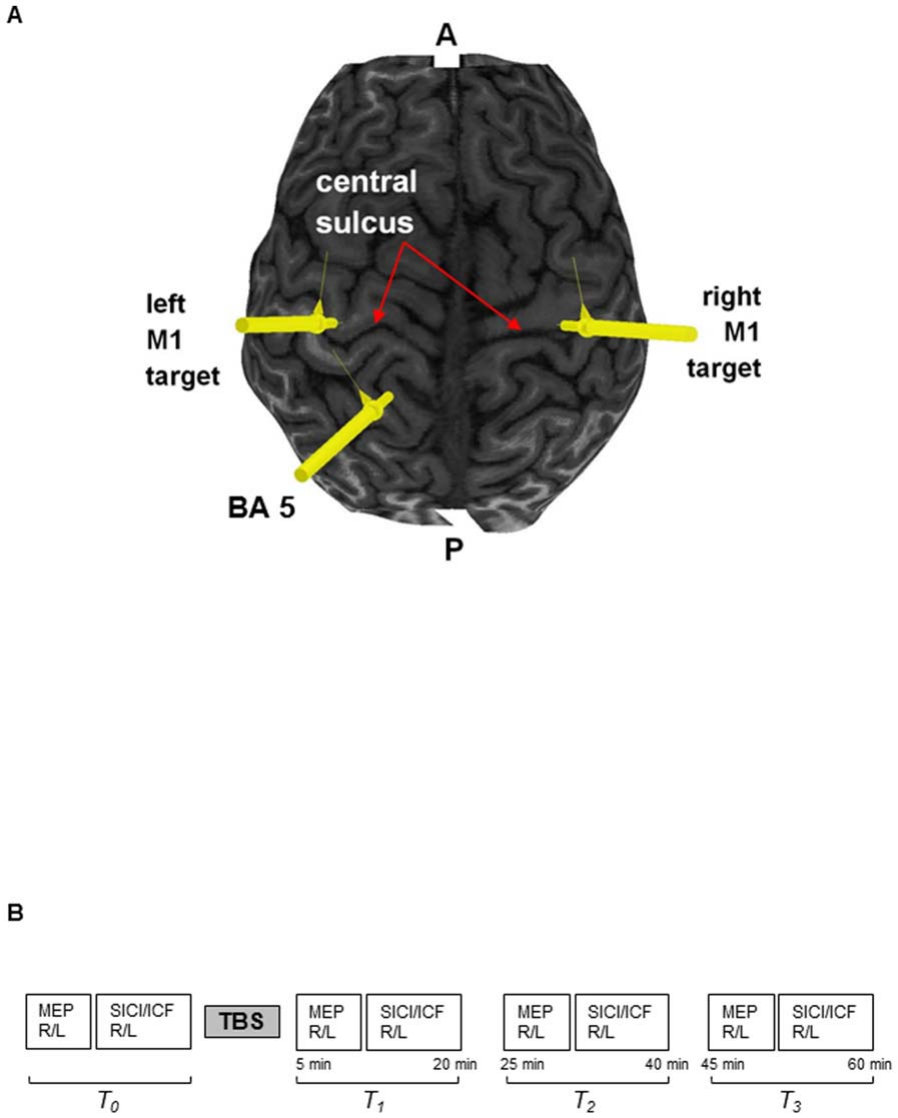
A. TMS target locations. MRI from one participant demonstrating the targets used for cTBS within BA 5 and M1 bilaterally. A (anterior), P (posterior). Yellow lines indicate location of TMS coil placement. B. Experimental Time Course. Graphic representation depicting the order of data collection and experiment procedures. RFDI/LFDI (right, left first dorsal interosseous), MEPs (motor evoked potentials), SICI (short interval intracortical inhibition), ICF (intracortical facilitation), TBS (theta-burst stimulation).

Single and paired-pulse magnetic stimulation were delivered using two custom built 50 mm inner diameter figure-of-eight branding coils connected to two Magstim 200^2^ stimulators (Magstim, Whitland, UK). Theta-burst stimulation (TBS) was applied using a 90 mm outer diameter figure of eight coil with a MagPro stimulator (MCF-B65; Medtronic, Minneapolis, MN, USA). To determine the motor hotspot for FDI in M1 of each hemisphere, a branding coil was positioned over left or right M1 and oriented 45 degrees to the mid-sagittal line to induce a current in the posterior to anterior direction. The motor hotspot was defined as the M1 location optimal for eliciting a motor evoked potential (MEP) in the contralateral relaxed FDI muscle. Active motor threshold (AMT) was determined at the motor hotspot and defined as the lowest intensity required to evoke MEPs of >200 µV amplitude in 5 out of 10 consecutive trials during 10% maximum voluntary contraction of FDI [37]. Brainsight Neuronavigation (Rogue Research, Canada) was used to align the location of the coils with respect to cortical targets using MRI data. MRI was conducted on a 3T GE scanner (172 images) with 3DFSPGR-IR sequences using a 20 cm FOV (256×256). The coils were held in place using coil holders mounted on the Brainsight Neuronavigation apparatus. In Brodmann's mapping of the superior parietal lobule, area 5 and 7 were positioned medial to the intraparietal sulcus, with area 5 extending medially to the midline of the brain and extending lateral and posterior to abut area 7. However, the boundary between Brodmann areas 5 and 7 in humans is not discernable using gross anatomy. We therefore defined BA 5 as the cortical territory occupying the medial SPL, medial to the intraparietal sulcus and posterior to the postcentral gyrus using the BA 5 boundaries outlined in the Talairach atlas [38] and referenced to the Brodmann illustration [39]. The TMS coil for BA 5 stimulation was positioned over SPL at 1.6 cm (±0.26) lateral to the midline of the brain using the MRI obtained from each participant. Figure 1A displays an example of the TBS location for one participant. For all experiments, measurements were obtained from the left and right FDI before and at 5–20 minutes, 25–40 minutes, and 45–60 minutes following TBS. The order of right versus left hemisphere stimulated was randomized across participants. Figure 1B displays a schematic of the experimental timeline.

### Experiment 1: CTBS over area 5 on MEPs, SICI and ICF

In 12 participants (mean age ± SD, 26±3.7) cTBS was applied over area 5 within the left hemisphere at 80% AMT using the 600 pulse protocol [6]–[8], [40]. The coil was positioned slightly medial (10 degrees) to induce a posterior to anterior directed current in the underlying tissue. MEPs, SICI and ICF were measured as depicted in Figure 1B. For MEPs, each time block consisted of 15 single TMS pulses applied over the left and right M1. TMS intensity was set at a value that evoked MEPs of ∼1 mV amplitude in LFDI and RFDI before cTBS and the same value was used following stimulation [7], [8]. For SICI and ICF, both the conditioning and test stimuli were applied over M1 through the same coil connected to a Magstim 200^2^ stimulator operating via a Bistim module. Paired-pulse paradigms, SICI and ICF, were performed using the previously published protocol [28] whereby a subthreshold conditioning stimulus (CS) is followed by a suprathreshold test stimulus (TS) to the FDI motor hotspot. The interstimulus interval (ISI) for SICI and ICF was 3 and 10 ms, respectively to achieve intracortical inhibition and facilitation [28], [41]. To measure SICI and ICF, a block consisted of TS alone, ISI of 3 ms (SICI) and ISI of 10 ms (ICF). Each ISI and TS alone trials were randomly presented 15 times during the block. The CS was set at 80% AMT for SICI and ICF as determined before cTBS stimulation and kept constant throughout the experiment [7], [8]. The TS intensity was adjusted to evoke MEPs in contralateral FDI of ∼1 mV before and after cTBS [6]–[8]. Stimulation intensities of the CS and TS were adjusted to accommodate the reduced output of the Bistim module. Fifteen trials with an inter-trial interval of 5 seconds were collected for left and right SICI and ICF. Twelve healthy subjects participated in the sham control (mean age ± SD, 22.1±2.56) and were positioned in the Brainsight apparatus with their surface skull anatomy aligned with a standard MRI. AMT was collected to determine CS intensities for SICI and ICF. For sham cTBS, the coil was positioned over an approximation of BA 5 and the sound of the coil was played without delivering any current, similar to the methods used elsewhere [42]. No subject reported knowing that the stimulation was a sham placebo. MEPs and SICI/ICF for the sham group were recorded at the same intervals as shown in Figure 1B.

### Experiment 2: ITBS over area 5 on MEPs, SICI and ICF

Eleven healthy, right-handed subjects (mean age ± SD, 27.3±3.66) received iTBS applied over area 5 within the left hemisphere at 80% AMT using the 600 pulse protocol [6]–[8], [33], [40]. The coil was positioned slightly medial (10 degrees) to induce a posterior to anterior directed current in the underlying tissue [6], [7]. MEPs and SICI/ICF were recorded using the same methodology and at the same time intervals as in Experiment 1.

### Experiment 3: Influence of cTBS over area 5 on spinal motor neuron excitability

To test the possibility that BA 5 influences MEPs via a spinal route, F-waves were measured in a subset of 7 participants (mean age ± SD, 28.17±4.95). F-waves were elicited by supramaximal stimulation of the ulnar nerve at the wrist (0.2 ms constant current pulse) [43] and surface EMG was recorded from the FDI muscle of the stimulated side. F- waves were recorded for both the right and left FDI muscles. Due to the variability in the persistence of the F-wave, one-hundred stimuli were delivered for the right and left sides and were collected in the four time blocks used in Experiments 1 and 2 (Figure 1B). To be deemed acceptable for further analyses, the amplitude of each F-wave must have been ≥50 µV. To obtain the mean F-wave amplitude for each time block, only the first 15 F-waves collected that met the amplitude criteria were averaged, similar to the number of trials used elsewhere [44]. To assess F-wave latency, the timing of the peak of the waveform was determined and averaged for the 15 F-waves that met the amplitude criteria.

### Data Analysis

Experiment 1 used two-way repeated measure analyses of variance (ANOVA) with between subject factor INTERVENTION (2 levels; TBS, sham TBS) and TIME (4 levels; pre, post block 1, post block 2, post block 3) for each dependent measure (SICI, MEP, ICF) for left and right FDI. Experiments 2 and 3 used a one-way repeated measure ANOVA using within-subject factor TIME (4 levels; pre, post block 1, post block 2, post block 3) for each dependent measure (Experiment 2: SICI, MEP, ICF for right and left FDI, Experiment 3: F-wave amplitude and latency measured in the right FDI and in the left FDI). Each ANOVA conducted passed the Greenhouse-Geisser and Huynh-Feldt tests for sphericity. Significance was set at p≤0.05.

## Results

### Experiment 1: CTBS over area 5

#### MEPs

**Figure 2:**
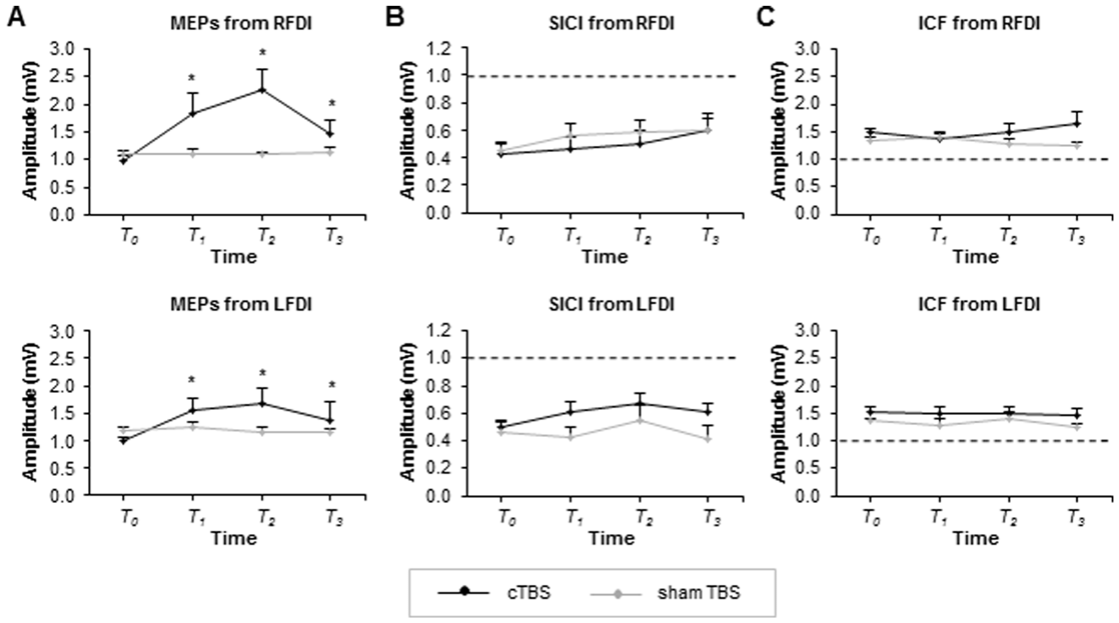
CTBS over area 5 on MEPs, SICI and ICF. A. Group-averaged MEPs (with standard errors) obtained from the right (top) and left (bottom) FDI for the cTBS group (black line) and sham group (gray line). B. Group-averaged SICI obtained from the right (top) and left (bottom) FDI for the cTBS group (black line) and sham group (gray line). C. Group-averaged ICF obtained from the right (top) and left (bottom) FDI for the cTBS group (black line) and sham group (gray line). Time course denoted as T_o_ (before TBS), T_1_ (5–20 min post TBS), T_2_ (25–40 min post TBS), T_3_ (45–60 min post TBS). *p≤0.05.

All participants successfully completed the experiment. The mean stimulator output used for delivery of cTBS was 38.17% (±7.8). The two-way ANOVA for MEPs recorded over right FDI, contralateral to BA 5 cTBS revealed significant main effects of INTERVENTION (F_(1,66)_ = 4.93, p = 0.037) and TIME (F_(3,66)_ = 7.76, p = 0.0002), and an interaction between INTERVENTION and TIME (F_(3, 66)_ = 7.96, p = 0.0001). Post-hoc Tukey's test revealed that MEP amplitude was significantly greater at 5 (p = 0.0001), 25 (p = 0.0001), and 45 (p = 0.0159) minutes following cTBS compared to pre-cTBS values. There were no differences amongst MEP amplitudes in the sham intervention. Figure 2A (top) plots the group-averaged data (with standard errors) for the MEPs obtained from right FDI. For MEPs recorded over left FDI, ipsilateral to BA 5 cTBS, the two-way ANOVA revealed no effect of INTERVENTION (F (1,66) = 1.04, p = 0.3185), a significant main effect of TIME (F (3,66) = 3.05, p = 0.0344), and an interaction that trended toward significance (INTERVENTION and TIME (F (3,66) = 2.62, p = 0.0583). Post-hoc Tukey's test revealed that compared to pre-TBS, MEP amplitude was significantly greater at 5 (p = 0.0026), 25 (p = 0.0003), and 45 (p = 0.0308) minutes following cTBS. Figure 2A (bottom) plots the group-averaged data (with standard errors) for the MEPs obtained from left FDI.

### SICI and ICF

The group averaged TS alone amplitudes during SICI and ICF (with standard errors) for pre, post block 1, 2 and 3 were 1.12 (0.07), 1.23 (0.05), 1.15 (0.07) and 1.11 (0.05), respectively for MEPs recorded over right FDI before and after cTBS. Similarly for sham TBS, group averaged TS alone amplitudes for right FDI MEPs for the respective blocks were 1.12 (.07), 1.08 (.06), 1.10 (.06) and 1.17 (0.06). The group averaged TS alone amplitudes (with standard errors) for left FDI for pre, post block 1, 2 and 3 were 1.01 (0.06), 1.02 (0.07), 1.06 (0.09) and 1.15 (0.04), respectively for the cTBS group. Similarly, for sham TBS, the group averaged TS alone amplitudes for left FDI were 1.04 (.05), 1.13 (.07), 1.05 (.05) and 1.15 (.06). For SICI recorded over right FDI, contralateral to BA 5 cTBS, the two-way ANOVA revealed no effect of INTERVENTION (F_(1,57)_ = 0.26, p = 0.6163), TIME (F_(3,57)_ = 2.30, p = 0.0868), and no interaction between the INTERVENTION and TIME (F_(3,57)_ = 0.31, p = 0.8210). For SICI recorded over left FDI, ipsilateral to BA 5 cTBS, the two-way ANOVA revealed no effects of INTERVENTION (F_(1,60)_ = 2.24, p = 0.1502), TIME (F_(3,60)_ = 2.67, p = 0.0554), or INTERVENTION and TIME (F_(3,60)_ = 0.99, p = 0.4043). Figure 2B displays the group-averaged SICI (with standard errors) for right (top) and left FDI (bottom) before and after cTBS and sham TBS.

ICF was unaltered following cTBS over area 5. No effects following cTBS were observed for ICF measured from the right (INTERVENTION (F_(1,57)_ = 3.79, p = 0.0664), TIME (F_(3,57)_ = 0.14, p = 0.9356) or INTERVENTION and TIME (F_(3,57)_ = 1.27, p = 0.2922)) or left (INTERVENTION (F_(1,54)_ = 2.69, p = 0.1183), TIME (F_(3,54)_ = 0.40, p = 0.7526), or INTERVENTION and TIME (F_(3,54)_ = 0.16, p = 0.9199)) FDI muscles. Figure 2C displays the group-averaged ICF (with standard errors) for right (top) and left FDI (bottom) before and after cTBS and sham TBS.

### Experiment 2: ITBS over area 5

#### MEPs

**Figure 3:**
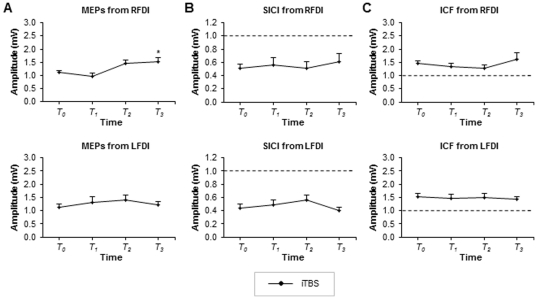
ITBS over area 5 on MEPs, SICI and ICF. A. Group-averaged MEPs (with standard errors) obtained from the right (top) and left (bottom) FDI for the iTBS group. B. Group-averaged SICI obtained from the right (top) and left (bottom) FDI for the iTBS group. C. Group-averaged ICF obtained from the right (top) and left (bottom) FDI for the iTBS group. Time course denoted as T_o_ (before TBS), T_1_ (5–20 min post TBS), T_2_ (25–40 min post TBS), T_3_ (45–60 min post TBS). *p≤0.05.

All participants successfully completed the experiment. The mean stimulator output used for delivery of iTBS was 36% (±6.9). For MEPs recorded over right FDI, contralateral to BA 5 iTBS, the one-way ANOVA revealed a significant effect of factor TIME (F_(3,30)_ = 6.84, p = 0.0012). Post-hoc Tukey's test revealed that compared to pre-theta-burst, MEP amplitude was significantly greater at 45 (p≤0.05) minutes following iTBS. There was no difference compared to pre-TBS at 5 or 25 minutes (p≥0.05). Figure 3A (top) displays the group-averaged MEPs (with standard errors) for right FDI before and after iTBS. For MEPs recorded over left FDI, ipsilateral to BA 5 iTBS, the one-way ANOVA revealed no effect TIME (F_(3, 30)_ = 0.47, p = 0.706). Figure 3A (bottom) displays the group-averaged MEPs (with standard errors) for left FDI before and after iTBS.

### SICI and ICF

The group averaged TS alone amplitudes (with standard errors) for pre, post block 1, 2 and 3 were 1.04 (.07), 1.01 (.06), 1.13 (.07) and 1.05 (.06) respectively, for MEPs recorded over right FDI. For left FDI the group averaged TS alone amplitudes (with standard errors) for each block respectively were 1.09 (.07), 1.13 (.10), 1.19 (.06) and 1.15 (.04). For SICI recorded from right FDI, contralateral to BA 5 iTBS, the one-way ANOVA revealed no significant main effect of TIME (F_(3, 21)_ = 0.47, p = 0.705). Similarly, for SICI recorded from left FDI, ipsilateral area 5 iTBS, the one-way ANOVA revealed no effect of TIME (F_(3, 27)_ = 1.44, p = 0.253). Figure 3B displays the group-averaged SICI (with standard errors) for right FDI (top) and left FDI (bottom) muscles, respectively, before and after iTBS.

ICF recorded over right FDI revealed no significant effect of TIME (F_(3, 27_ = 1.78, p = 0.175). Similarly, for left FDI there was no effect of TIME (F_(3, 30)_ = 0.27, p = 0.849). Figure 3C displays the group-averaged ICF (with standard errors) for right (top) and left FDI (bottom) muscles, respectively, before and after iTBS.

### Experiment 3: Influence of cTBS over area 5 on spinal motor neuron excitability

All participants successfully completed the experiment. The one-way ANOVA for the F-wave amplitude revealed no significant effect of TIME for the right FDI (F_(3, 18)_ = 1.11, p = 0.3708) and left FDI (F_(3, 18)_ = 0.54, p = 0.6624). The mean F-wave amplitudes recorded from right FDI were .21, .23, .20, .21 mV and for left FDI were .18, .18, .19, .19 mV for T_o_, T_1_, T_2_, T_3_, respectively. The latency of F-waves were also unchanged following cTBS (right FDI mean latency of 31.4, 32.0, 32.2, and 32.5 ms for T_o_, T_1_, T_2_, T_3_, respectively, F_(3, 18)_ = 2.59, p = 0.09; left FDI mean latency of 31.7, 31.7, 32.6, 32.0 ms for T_o_, T_1_, T_2_, T_3_, respectively, F_(3, 18)_ = 1.29, p = 0.31).

## Discussion

The experiments presented are the first investigation of the influence of BA 5 on neural circuitry within M1 in humans. We focused on the influence of BA 5, located in the medial superior parietal lobule, on the M1 neural circuitry related to muscles of the hand. This area is suggested to have evolved with the ability to perform skilled hand manipulation [11] and may provide important neural signals to modify the cortical output to hand muscles. We assessed MEPs, ICF, and SICI from right and left FDI before and for up to one hour following cTBS, iTBS and sham TBS applied over BA 5. A subsequent experiment investigated spinal motor neuron excitability by measuring F-waves from the right and left FDI muscles before and following cTBS over BA 5. Novel observations include an increase in MEPs bilaterally for up to one hour with amplitude changes that exceed those observed when cTBS is applied directly over M1 [6]. Surprisingly, iTBS had effects similar to cTBS and increased MEPs in the contralateral FDI for a comparable duration. However, M1 circuitry mediating SICI and ICF, and spinal motor neuron excitability as measured with F-waves were unaltered by TBS to BA 5. Collectively, these data suggest that BA 5 modulates M1 excitability directed to the hand and likely via interaction with the corticospinal neurons within M1.

MEP amplitude increased following cTBS applied over left hemisphere BA 5. At first glance, these findings are surprising since MEP amplitude decreases following cTBS directly over M1 [6], [7], [32] and the premotor cortex [8]. However, in these latter investigations, cTBS is applied over loci dominated by motor functions and it may be that cTBS over sensory or sensorimotor areas may yield differing effects on excitability, as suggested elsewhere [45]. For example, cTBS to left primary somatosensory cortex (SI) does not decrease or increase MEPs [33].

CTBS over BA 5 versus SI yield differing after-effects on M1 cortical excitability. We observed an increase in cortical excitability following cTBS over BA 5. In contrast, cTBS over SI does not modulate MEP amplitude [33]. These differences may reflect the unique functional contributions to processing within M1 such as a dominant role for BA 5 in motor control of the hand [9] and a sensory feedback role for SI. Alternatively, the differences in cTBS effects may relate to the density of projections from BA 5 versus SI to M1. Studies in monkeys reveal an equal or greater density of projections from BA 5 to M1 compared to projections from SI [15], [18]. Last, while cortical magnification exists in SI and BA 5, the latter area is almost entirely dedicated to the representation of the hand and forelimb [10] suggesting that BA 5 may have a critical role in influencing M1 neural circuitry and output specifically directed to the hand.

CTBS and iTBS directed to M1 lead to a decrease and increase in MEP amplitude, respectively [7], [32]. Similar opposite effects are observed following cTBS and iTBS over the cerebellum [46]. The opposite effects of TBS protocols are also observed by measuring somatosensory evoked potentials (SEPs); SEPs are increased following iTBS [40], [47] and decreased following cTBS [33]. In contrast to these findings, the present study revealed that both TBS protocols increased MEPs when applied over BA 5, an effect that may be specific to the region of sensory cortex receiving TBS. For example, following iTBS or cTBS over SI, the amplitude of laser-evoked potential N2 recorded from secondary somatosensory cortex is decreased for both TBS protocols [48]. Similarly, iTBS versus cTBS over visual cortex do not yield opposite effects on perception; cTBS increases phosphene threshold while iTBS has no effect [49]. One explanation for the similar pattern of iTBS and cTBS results may relate to the timecourse of induced neuronal effects within somatosensory cortex. When equivalent repeats of cTBS and iTBS are delivered to rat cortex, both protocols yield an increase in gamma power EEG and multi-unit action potentials recorded within SI during the hour of stimulation [50]. For these measures, the differing effects of the two protocols were only observed several hours following stimulation [50]. Although it is difficult to make direct comparisons with the present human work, these data suggest that iTBS and cTBS may indeed induce similar neural changes in sensory cortex.

BA 5 selectively influenced M1 excitability such that MEPs were increased while SICI and ICF were unchanged, a finding similar to the effects following TBS over the premotor cortex [8]. Changes in MEPs and not SICI or ICF would occur if BA 5 influences corticospinal neurons (CSN) within M1 or spinal motor neuron excitability without changing the excitability of interneurons involved in SICI and ICF. In support of a CSN mechanism, retrograde and anterograde labelling demonstrate BA 5 projections to output neurons within M1 [15], [18]. Anatomical labelling also reveal a direct axonal projection from BA 5 to the dorsal horn of the spinal cord [47], [48] that could potentially modulate the spinal output neurons in the ventral horn. However, we observed that F-wave amplitude remained unaltered following cTBS suggesting that BA 5 most likely influences M1 output at a cortical and not at a spinal level, similar to the explanation for the effects of TBS over M1 where H-reflexes remain unaltered [6]. However, it may be that the F-wave probes distinct motor neuronal pools separate from those activated by the TS pulses applied over M1. If true, increases in MEP amplitude may occur without changes to the F-wave amplitude. Future studies may probe other spinal circuits such as H-reflexes using reciprocal inhibition to gain further insight into the spinal influence of BA 5. Alternatively, BA 5 may influence M1 output indirectly via other cortical loci. BA 5 has dense projections to the premotor cortex and the supplementary motor area both of which project to M1 [18] via the SLF [26]. The observation that MEPs increase ipsilateral to TBS suggests that transcallosal connectivity between homologous BA 5 [11] or M1 cortices may be modulated.

TBS over remote areas may be more effective at driving changes in M1 than TBS applied directly over M1. Following cTBS, the maximal change we observed in right FDI MEP amplitude was 132%, well exceeding the 42.4% difference seen following cTBS over M1 [6]. However, iTBS over BA 5 lead to smaller maximal amplitude changes compared to iTBS over M1 (38.3% versus 75.7%) [6]. Similar amplified effects are observed following TMS to the premotor cortex. Using rTMS over premotor cortex and not M1, MEP amplitudes increased ∼60% (Figure 2A in [51]). Further, cTBS over premotor cortex leads to longer lasting changes that build up and become more robust than alterations following cTBS applied directly to M1 [8]. It has been suggested that TMS protocols applied to premotor and anterior cortical loci may have a stronger impact on M1 cortical excitability than those applied directly to M1 [52]. We extend this suggestion to include the medial SPL, BA 5, that provides a powerful and long-lasting modulation of M1 cortical excitability bilaterally. The differing influence induced by TBS over M1 versus remote areas projecting to M1 may relate to different mechanisms by which the protocol acts within these areas. One possibility is that TBS over BA 5 induces sustained changes in the activity of neurons projecting to M1, which in turn, could act to modify the background activity of M1 neurons. For example, cortical cooling of the secondary somatosensory cortex can reduce the background activity in SI neurons, an effect thought to be mediated by removal of a background facilitatory influence [53]. Some methodological factors require consideration and may influence the interpretation of the present results. The observed effects may relate to the intensity and direction of TBS current direction [7], [54]. TBS was delivered at 80% AMT with the induced current flowing in the posterior to anterior direction within the cortex. It may be that lower intensities would yield changes in SICI as observed following cTBS over M1 [55] and that currents induced in other directions would induce changes in the contralateral but not ipsilateral FDI [7]. Further, it remains unclear whether the observed effects are specific for BA 5 in the left hemisphere or can be seen following TBS to the homologous area in the right hemisphere. Functional MRI demonstrates differences between the right and left SPL during the stages of tactile object discrimination [24].

BA 5 provides a novel and alternative means to modify select M1 neural circuitry. Predictions can be made regarding the translation of these findings to the actual control of hand movement. For example, TBS to BA 5 may modulate the posture of the hand during reach to grasp tasks. In monkeys, 83% of area 5 neurons increased their firing during the posturing of the fingers prior to object grasp [56]. BA 5 neurons are sensitive to spatial kinematics such as position, direction, and displacement of the upper limb [9], [56] suggesting that these attributes of control may be manipulated following TBS over BA 5. Further, the performance of motor tasks expected to recruit BA 5 neurons such as skilled hand manipulation and thumb opposition movements may be altered following TBS. The present findings indicate that TBS over BA 5 induces robust changes in M1 cortical excitability in healthy individuals. Abnormalities within the M1 neural circuitry is observed in neurological populations such as stroke [5] and focal hand dystonia [2], [3]. Such circuitry may be modulated by TBS applied to areas remotely connected with M1. For example, cTBS over dorsal premotor cortex increases SICI and improves writing speed in Writer's cramp focal hand dystonia [57]. Future studies may examine the potential for BA 5 to modulate the abnormal levels of M1 excitability in such populations in an attempt to alter imbalances in the circuitry mediating hand control.
